# Acute fibrinous and organizing pneumonia complicated with hemophagocytic lymphohistiocytosis caused by chronic active Epstein-Barr virus infection: a case report

**DOI:** 10.1186/s12879-021-06868-0

**Published:** 2021-12-04

**Authors:** Xiaojing Wu, Kejing Wang, Yayue Gao, Ying Cai, Wenqiao Wang, Dingrong Zhong, Qingyuan Zhan

**Affiliations:** 1 Department of Pulmonary and Critical Care Medicine, Center of Respiratory Medicine, China-Japan Friendship Hospital, National Clinical Research Center for Respiratory Diseases, No 2, East Yinghua Road, Chaoyang District, Beijing, 100029 China; 2 Department of Second Senior Ward, China-Japan Friendship Hospital, Beijing, China; 3 Department of Hematology, China-Japan Friendship Hospital, Beijing, China; 4 Department of Pathology, China-Japan Friendship Hospital, Beijing, China

**Keywords:** Chronic active Epstein-Barr virus infection, Acute fibrinous and organizing pneumonia, Hemophagocytic lymphohistiocytosis, Consolidation in lung

## Abstract

**Background:**

Acute fibrinous and organizing pneumonia (AFOP) is a rare lung condition that is associated with acute lung injury. Its etiology may be idiopathic or secondary to a series of conditions, including immune-related diseases, unclassified connective tissue diseases, hematopoietic stem cell transplantation, infections, hematological diseases and drug induced lung toxicity. We report for the first time a case of AFOP complicated with hemophagocytic lymphohistiocytosis (HLH) caused by chronic active Epstein-Barr virus (CAEBV) infection.

**Case presentation:**

A 64-year-old man was admitted with a complaint of fever and dyspnea for 2 weeks. The patient presented with elevated serum aminotransferase levels, splenomegaly, progressive decrease of red blood cells and platelets, hyperferritinemia, hypofibrinogenemia, and elevated of Soluble interleukin-2 receptor (sCD25). His chest computed tomography (CT) scan revealed multiple patchy consolidation in both lungs and multiple lymphadenopathy in the mediastinum and hilum. The serology for antibodies of VCA-IgG was positive, EBV-DNA in peripheral blood was elevated, and EBV nucleic acid was detected in the alveolar lavage fluid. Histopathology of the lung tissue showed a dominant of intra-alveolar fibrin and organizing pneumonia. Hemophagocytic cells was found in the bone marrow smear and biopsy. EBV-DNA was detected in lung tissue and bone marrow using in situ hybridization with an EBV-encoded RNA (EBER) probe. After 50 days of hospitalization, he was improved in lung and hemogram.

**Conclusion:**

We report a case of AFOP with HLH caused by CAEBV in an immunocompetent adult, suggesting that AFOP may be a rare but serious complication caused by CAEBV, and glucocorticoid therapy may improve short-term prognosis.

## Background

Acute fibrinous and organizing pneumonia (AFOP) is a rare lung condition that is associated with acute lung injury. Its etiology may be idiopathic or secondary to a series of conditions, including immune-related diseases, unclassified connective tissue diseases, hematopoietic stem cell transplantation, infections (bacteria, fungi, viruses, etc.), hematological diseases and drug induced lung toxicity [1]. Many viral infections, including H1N1 [2], HIV [3] and coronavirus [4] have been reported to be associated with AFOP. Epstein-Barr virus (EBV) is one of the most common viruses in the human body without causing disease symptoms. In recent years, the development of molecular, biological and immunological detection methods makes it possible to detect viral specific genes in affected tissues. The highly sensitive and specific detection methods for EBV have enabled investigators to delineate the role of the EBV in various diseases of unknown origin [5, 6]. We report for the first time to our knowledge a case of AFOP complicated with hemophagocytic lymphohistiocytosis (HLH) caused by chronic active EBV (CAEVB) infection. Both the genomic and traditional examination approaches were used in the diagnosis of the reported case.

## Case presentation

In September 25, 2018, a 64-year-old man was admitted into our hospital with a complaint of fever, dry cough and dyspnea for 2 weeks while the patient was on antibiotic treatment for pneumonia. In July 6, 2016, the patient was hospitalized due to fever and multiple lymphadenopathy. At that time, an EBV-DNA load of 2.8*10^4^ copies/ml was detected in the patient’s plasma. After 2 weeks of hospitalization, the enlarged lymph nodes subsided and the patient was improved without specific treatment. The patient also had a history of type 2 diabetes mellitus.

**Fig. 1:**
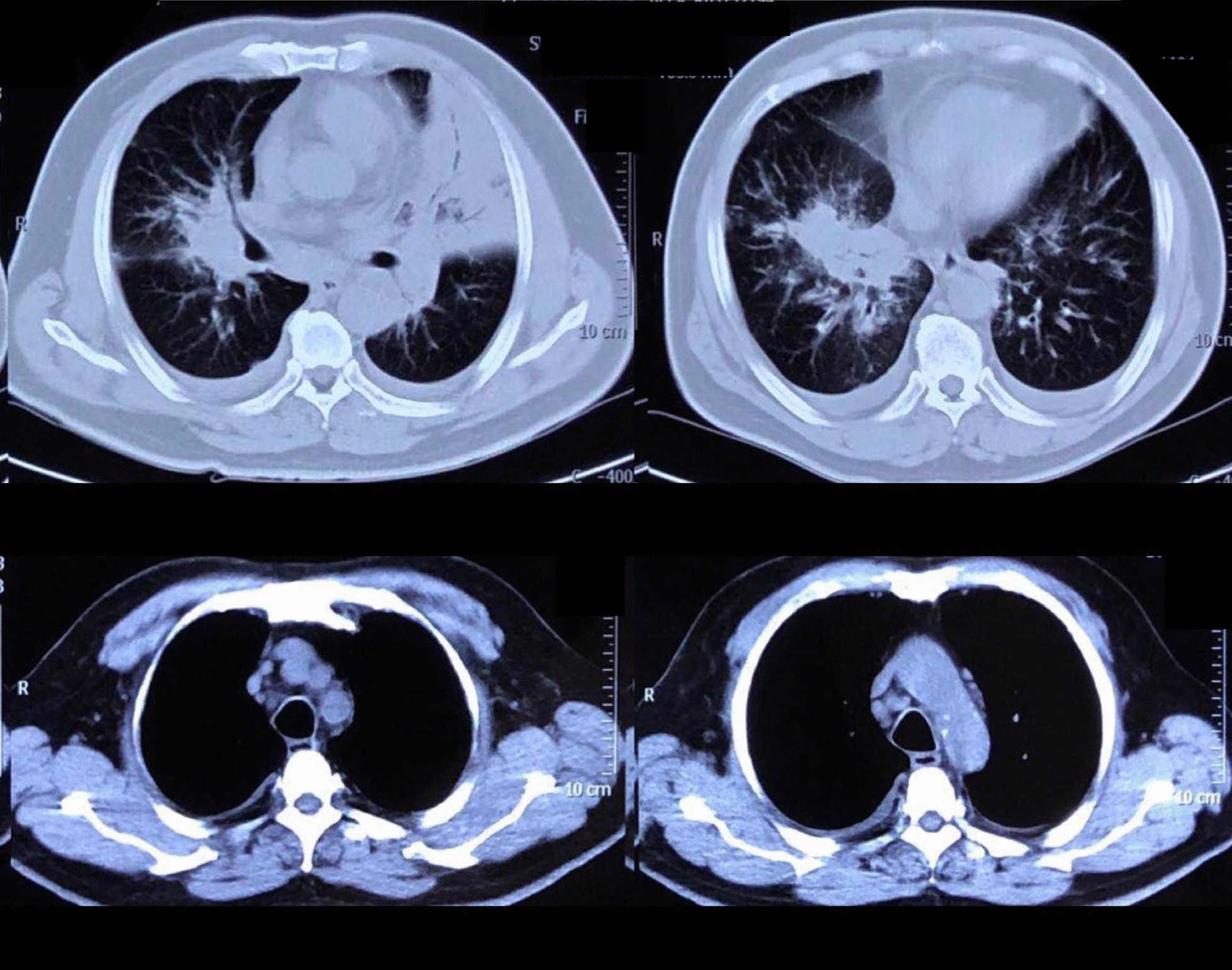
CT scan on admission: multiple patchy opacities with consolidation in both lungs, marked lesions in the left lung, and multiple lymphadenopathy in the mediastinum and hilum

On admission, his temperature was 39.4 °C; respiration rate was 30/min; blood pressure was 153/76 mmHg; pulse rate was 102/min; SpO_2_% was 88% with 45% high-flow nasal oxygen, PaO_2_/FiO_2_ was 135 mmHg; and wheezing was present in both lungs. Blood tests (Table 1) yielded a leukocyte count of 8.42 × 10^9^/L, hemoglobin level of 129 g/L, platelet count of 212 × 10^9^/L, procalcitonin level of 0.56 ng/ml, erythrocyte sedimentation rate of 39/mm, C-reactive protein level of 17.4 mg/dl, alanine aminotransferase (ALT) level of 93 IU/L, and aspartate aminotransferase (AST) level of 186 IU/L. The LDH of pleural fluid was 925 mmol/L. Serology for antibodies of VCA-IgG was 87.34RU/ml (< 16RU/ml), VCA-IgM was 0.08S/CO (< 0.8S/CO), VCA-IgA was 0.13S/CO (< 0.8S/CO) and EBV-DNA load in plasma was 3.27 × 10^4^ copies/ml. His chest computed tomography (CT) scan showed multiple patchy opacities with consolidation in both lungs, marked lesions in the left lung, and multiple lymphadenopathy in the mediastinum and hilum (Fig. 1). EBV nucleic acid in the bronchoalveolar lavage fluid (BALF) was positive by polymerase chain reaction (PCR). Other pathogenic microbials were not detected in the blood, urine, pleural effusion, or BALF. Piperacillin/tazobactam and levofloxacin were used as empirical antibiotics.

**Table 1:** Laboratory data

Complete blood cell count (on admission)		Serum chemistry	
White blood cell (× 10^9^/L)	8.42	ALT (IU/L)	93
Neutrophil (× 10^9^/L)	7.89	ALT (IU/L)	186
Eosinophil (× 10^9^/L)	0	Albumin (g/dl)	29.3
Basophil (× 10^9^/L)	0.02	T-Bil (μmol/L)	13.04
Monocyte (× 10^9^/L)	0.09	ALP (IU/L)	269
Lymphocyte (× 10^9^/L)	0.42	LDH (U/L)	894
Hemoglobin (g/dl)	129	BUN (mmol/L)	8.18
Platelet (× 10^9^/L)	212	Crea (μmol/L)	70.8
Procalcitonin (ng/ml)	0.56	K (mmol/L)	3.3
C-reactive protein (mg/dl)	17.4	Na (mmol/L)	130
Erythrocyte sedimentation rate (mm/h)	39	Triglyceride (mmol/L)	2.29
Complete blood cell count (10 days later)		sCD25 (pg/ml)	15,185
White blood cell (× 10^9^/L)	3.5	Serum ferritin (ng/ml)	> 15,000
Neutrophil (× 10^9^/L)	3.15	EB virus related test	
Hemoglobin (× 10^9^/L)	87	VCA-IgG	+
Platelet (× 10^9^/L)	32	EBV nucleic acid in BALF	+
Fibrinogen (g/L)	1.47	EBV-DNA (copies/ml)	3.27 × 10^4^

**Fig. 2:**
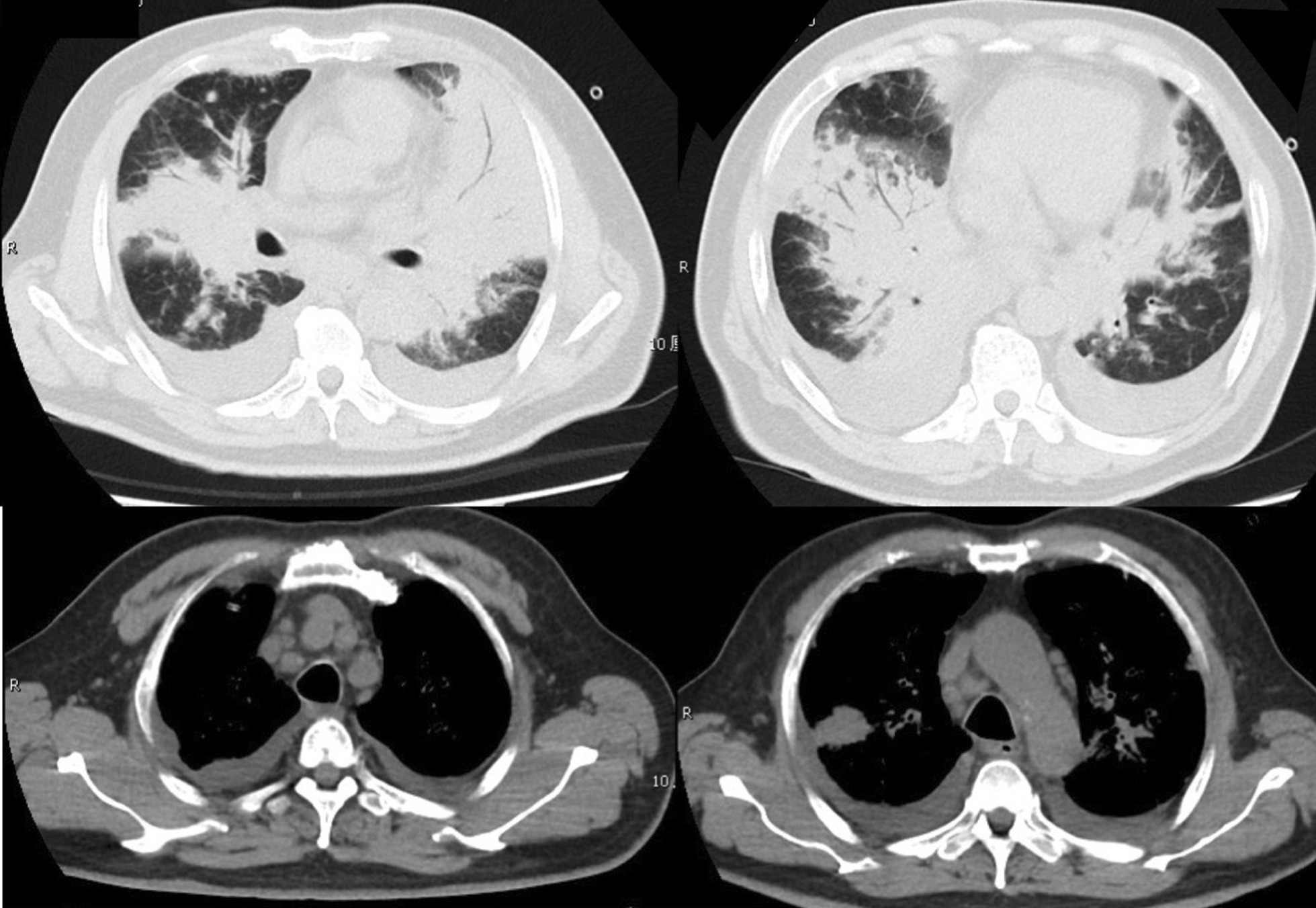
CT scan on day 10 after admission: both the consolidation and pleural effusion were exacerbated significantly

**Fig. 3:**
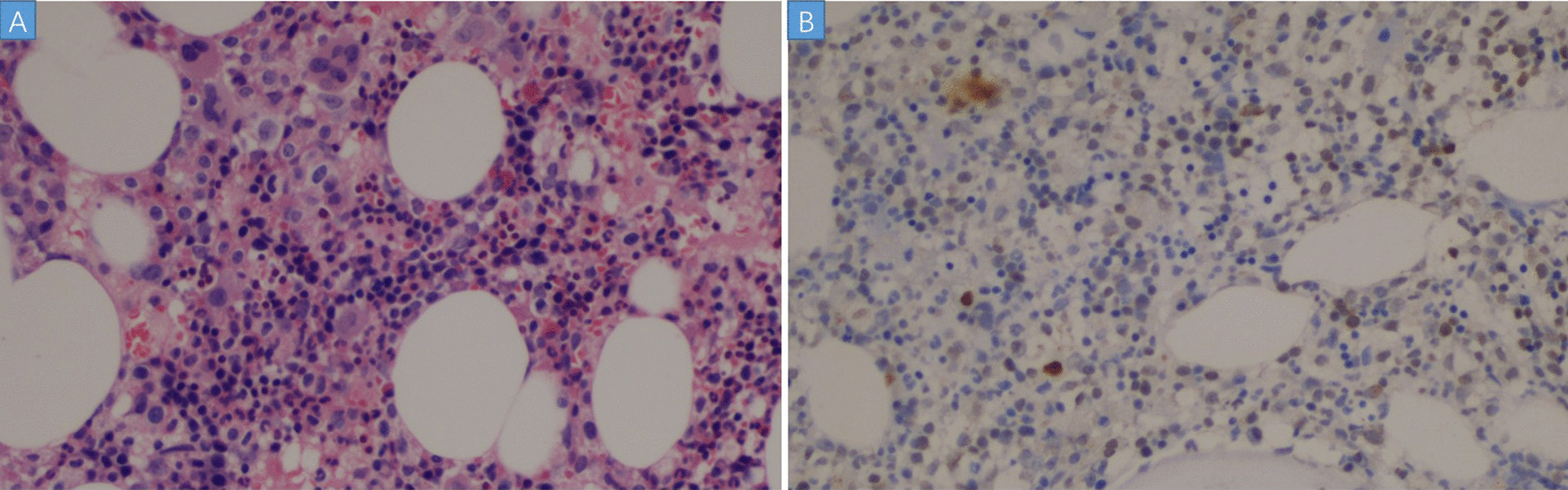
**A** Bone marrow biopsy showed a slight increase in granulocytes, which were scattered by lymphocyte infiltration, and the phenomenon of histocyte phagocytosis (hemophagocyte). (HE, × 200). **B** In situ hybridisation for EBERs shows EB virus-infected cells. (In situ hybridization EBERs, × 100)

**Fig. 4:**
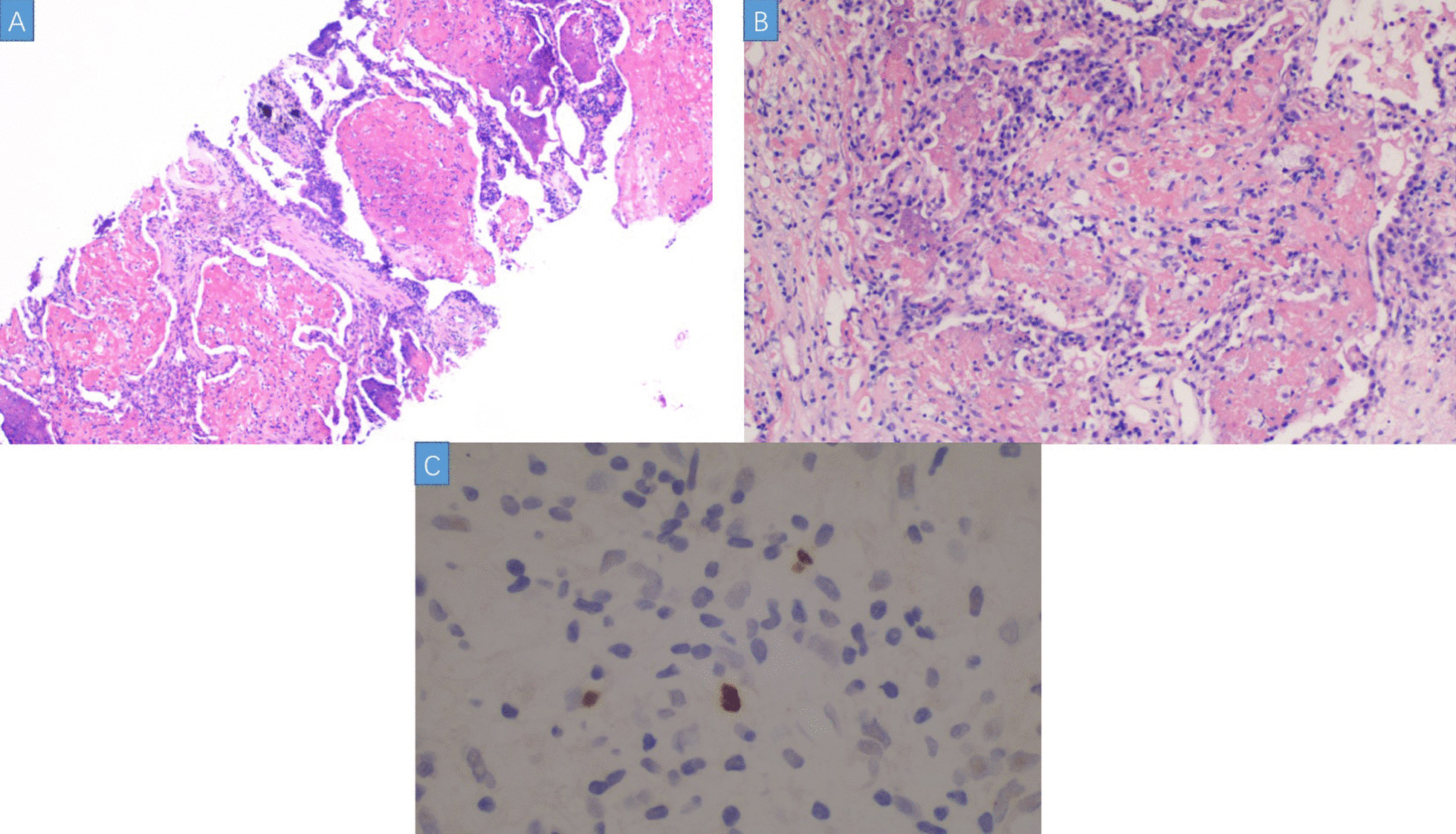
**A** Lung biopsy tissue showed a large number of fibrin were filled in the alveolar cavity, accompanied by acute inflammatory cells. There were no hyaline membranes or pulmonary edema. (HE, × 40). **B** Myofibroblasts proliferated in some alveolar cavities, showing the change of organic pneumonia. (HE, × 200). **C** EBV-positive activated lymphocytes in pulmonary interstitium. (In situ hybridization EBERs, × 200)

In 10 days after admission, the patient was till suffered fever and transferred to an intensive care unit where an intubation was required because of severe hypoxia. Meanwhile, a chest CT scan showed significant exacerbation (Fig. 2), and blood tests (Table 1) found a leukocyte count of 3.5 × 10^9^/L, hemoglobin level of 87 g/L, platelet count of 32 × 10^9^/L, fibrinogen of 1.47 g/L, triglyceride of 1.75mmo/L, serum ferritin level of > 15000 ng/ml, and soluble IL-2Ra (sCD25) level of 15185 pg/ml. Ultrasound showed splenomegaly with a length of 13 cm. In addition, a bone marrow examination identified hemophagocytosis. EBV DNA was detected in bone marrow using in situ hybridization with an EBV-encoded RNA (EBER) probe (Fig. 3). We used the SCOP 1 microscopy of Zeiss and MShot Image Analysis System was used to capture the microscopy images. Histopathology examination of a piece of lung tissue through percutaneous needle biopsy showed a dominant of intra-alveolar fibrin and organizing pneumonia, which suggested AFOP. In addition, EBERs were detected in the lung tissue (Fig. 4).

**Fig. 5:**
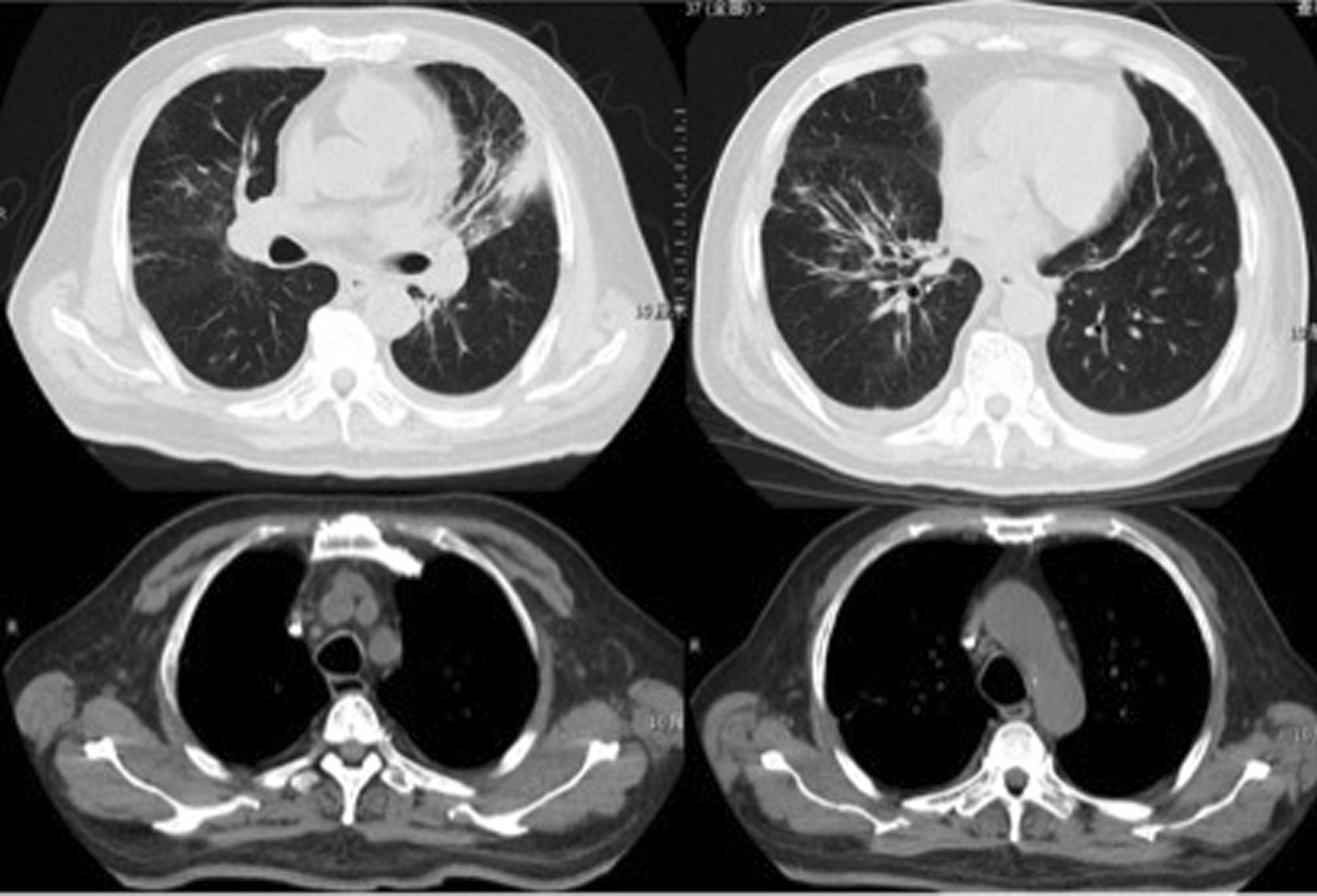
Chest CT scan on day 50 of hospitalization: lesions in both lungs were obviously absorbed, and the enlarged lymph nodes in the mediastinum and hilum were reduced or disappeared

Based on the above diagnoses, methylprednisolone at a dosage of 120 mg/d was administered for 5 days, followed by dexamethasone at a dosage of 10 mg/m^2^/d for 10 days and immunoglobulin at 0.4 g/Kg/d for 5 days. The fever that lasted for one month finally improved after methylprednisolone. Sulfanilamide and ganciclovir were used as prophylactic for fungal and virus. Two weeks later, the ventilator was able to be removed from this patient. In 50 days after admission, lung lesions were absorbed (Fig. 5), EBV-DNA load in plasma decreased to 2.2 × 10^2^ copies/ml, and the patient was discharged.

## Discussion and conclusions

This is the first case report of AFOP with HLH caused by CAEBV to our knowledge. HLH is a group of clinical syndromes characterized by multiple factors causing cytokine cascade release and tissue cell proliferation accompanied by phagocytosis of various hematopoietic cells. HLH is often secondary to infection, tumors and/or autoimmune diseases. Infection-related HLH is common, of which EB virus infection accounts for the majority. Tumor-related HLH is also common, especially in hematological malignancies such as lymphoma [7]. This patient had fever, splenomegaly, cytopenia, hypofibrinogenemia, hemophagocytic cells found in the bone marrow, significantly elevated serum ferritin and sCD25, meeting 7 of the 8 diagnostic criteria [8]. Therefore, the HLH diagnosis is clear.

The main clinical manifestation of AFOP is dyspnea, which may be accompanied by fever and cough. It is acutely or sub-acutely onset. Most acute cases develop respiratory failure rapidly, requiring mechanical ventilation treatment. The imaging manifestations of AFOP are mostly nodules and solid changes with unclear boundary in both lungs, mainly in the basal part and/or along the peribronchial vascular distribution [9]. Histopathological features include large amount of cellulose deposition in alveolar cavity accompanied by organic loose connective tissue, lack of typical transparent membrane formation in diffuse alveolar injury, no granuloma, and no obvious eosinophil infiltration [10]. The clinical symptoms, imaging characteristics and histopathological features of our patient were consistent with those of AFOP, so the diagnosis of AFOP was clear. AFOP can be secondary to viral infection, bacterial infection, connective tissue diseases and so on. This patient had subacute onset, and the formation of AFOP may be related to the acute exacerbation of CAEBV. The overall prognosis of AFOP patients is poor, and the mortality rate can be as high as 50%. Some cases respond well to glucocorticoids and immunosuppressive agents in the acute phase, but relapse in the course of hormone reduction, and their long-term prognosis is still poor [9]. Our patient was treated with methylprednisolone before taking HLH into account. Glucocorticoids dosage was then given according to the HLH treatment guideline.

EBV is usually a latent infection in the human body, and some patients may develop into chronic active infection [11]. T-cell Chronic active Epstein-Barr virus tends to predominate in Asia, while B-cell CAEBV is frequently documented in Western regions. The differential diagnosis between CAEBV and hemophagocytic lymphohistiocytosis (EBV-HLH) is extremely challenging from clinical and pathologic points of view. Guidelines for classification between EBV-HLH and CAEBV are not clear and the two definitions are used interchangeably by various authors [12–14]. The diagnostic criteria of CAEBV include: (1) Persistent or recurrent IM-like symptom including fever, swelling of lymph nodes, and hepatosplenomegaly; (2) Unusual pattern of anti-EBV antibodies with raised anti-VCA and anti-EA, and/or detection of increased EBV genomes in affected tissues, including the peripheral blood; (3) Chronic illness which cannot be explained by other known disease processes at diagnosis [15]. This patient had recurrent fever with lymphadenopathy at both the current episode and 2 years ago, significantly elevated EBV-DNA in peripheral blood, and EBERs in bone marrow and lung tissue. So, CAEBV diagnosis was clear. CABEV can lead to life-threatening complications, including HLH, DIC, liver failure, coronary artery aneurysm, central nervous system involvement, myocarditis, lymphoma and hematological malignancies [5, 16]. Few cases involving the lungs have been reported. Pulmonary diseases associated with EBV infection reported in the literature include hilar/mediastinal lymph node enlargement, pleural effusion and interstitial pneumonia [17]. CAEBV infection with pulmonary parenchymal involvement is rare in immunocompetent patients. Joo EJ et al. reported a case of interstitial pneumonia caused by CAEBV [18]. In that case, a 28 year old female was admitted because of fever and bilateral pleural effusion. Her clinical features are similar to those of our patient, including pancytopenia, liver dysfunction, splenomegaly, and multiple lymphadenopathy. Unlike our patient’s multiple patchy opacities with consolidation in both lungs, her chest CT showed diffuse interstitial lesions. She was also admitted to the ICU because of severe respiratory failure. Her lung histopathology showed alveolar septal lymphocyte infiltration. Jacek Roliński et al. reported a case of CAEBV complicated with interstitial pneumonia, which was considered to be associated with infectious diseases [19].

In conclusion, we report a case of AFOP with HLH caused by CAEBV in an immunocompetent adult, suggesting that AFOP may be a rare but serious complication caused by CAEBV, and glucocorticoid therapy may improve short-term prognosis.

## Data Availability

The datasets analyzed during the current study are available from the corresponding author on reasonable request.

## Abbreviations

AFOP: Acute fibrinous and organizing pneumonia
EBV: Epstein-Barr virus
HLH: Hemophagocytic lymphohistiocytosis
CAEBV: Chronic active EBV
ALT: Alanine aminotransferase
AST: Aspartate aminotransferase
CT: Computed tomography
BALF: Bronchoalveolar lavage fluid
EBER: EBV-encoded RNA
